# Soil-Nutrient Depletion and Microbial Community Restructuring in Continuous Celery Cropping: Opposing Responses of Bacteria and Fungi

**DOI:** 10.3390/biology15100771

**Published:** 2026-05-12

**Authors:** Junkang Sui, Na Wang, Hongliang Wang, Yanjie Li, Junlong Wang, Mengyao Duan, Mei Liao, Yuting Jiang, Xingang Zhou

**Affiliations:** 1 College of Horticulture, Northeast Agricultural University, Haerbin 150006, China; 2 College of Agriculture and Biology, Liaocheng University, Liaocheng 252000, China; 3 Shandong Binnong Technology Co., Ltd., Binzhou City 256600, China

**Keywords:** continuous cropping, celery, rhizosphere microbiome, soil nutrient imbalance

## Abstract

Continuous cropping obstacles represent a major constraint in agricultural production, yet the underlying microbial mechanisms remain incompletely understood. This study systematically compared soil physicochemical properties, microbial diversity, community composition, and nutrient-microbe relationships between continuous cropping (CC) and non-continuous cropping (CK) celery rhizospheres using high-throughput sequencing, soil physicochemical analysis, and Mantel tests. The results revealed that CC soils exhibited severe depletion of available potassium (AK, 69.9% decreased) and alkali-hydrolyzable nitrogen (AN, 65.9% decreased), accompanied by a modest but statistically significant accumulation of total phosphorus (TP, 8.0% increased). Strikingly, bacterial and fungal communities displayed diametrically opposed diversity responses: CC significantly reduced bacterial α-diversity (Shannon: 5.66 vs. 6.67, *p* < 0.01) and richness (ACE: 2018 vs. 2623, *p* < 0.01), whereas fungal diversity and richness more than doubled under CC (ACE: 619 vs. 296, *p* < 0.01; Shannon: 4.13 vs. 3.34, *p* < 0.01). β-diversity analyses (NMDS and ANOSIM) confirmed fundamental community restructuring in CC soils for both microbial domains. At the taxonomic level, CC soils showed significant depletion of beneficial plant growth-promoting rhizobacteria (PGPR), including *Bacillus* (↓89.3%), *Mesobacillus* (↓72.8%), and *Pseudomonas* (↓30.8%), coupled with dramatic enrichment of the phytopathogenic genus *Fusarium* (10.9-fold increase, 8.81% vs. 0.81%, *p* < 0.001). LEfSe analysis identified *Fusarium*, *Arrhenia*, and *Mortierella* as specific biomarkers of CC soils, whereas *Bacillus*, *Mesobacillus*, *Cladosporium*, and *Alternaria* were biomarkers of CK soils. Mantel tests further revealed that CC significantly altered nutrient-microbe coupling relationships, with bacterial communities significantly correlated with TP, AN, and OC, and fungal communities with TP, TK, AP, and AN. Collectively, these findings demonstrate that continuous celery cropping shifts the rhizosphere microbiome from a bacterial-dominated profile associated with beneficial taxa (e.g., *Bacillus*, *Pseudomonas*) toward a fungal-enriched profile dominated by the pathogen Fusarium, suggesting a potential transition from a putatively disease-suppressive to a disease-conducive microbial state.

## Simple Summary

Celery is a popular vegetable often grown continuously in the same soil to meet market demand. However, this practice can lead to “soil sickness,” where soil quality declines and plants become less healthy over time. In this study, we explored the hidden changes in the soil, especially the microscopic community of bacteria and fungi living around celery roots, after four consecutive cropping cycles. We found that continuous cropping severely depleted key nutrients in the soil, particularly nitrogen and potassium. Surprisingly, while the diversity and abundance of beneficial bacteria decreased sharply, the diversity of fungi more than doubled. Notably, a group of bacteria known to promote plant growth (*Bacillus*) was largely eliminated, while the population of a fungus group that includes the pathogen causing celery wilt (*Fusarium*) increased more than tenfold. These findings suggest that continuous cropping flips the soil’s microbial balance from a bacteria-friendly, potentially health-promoting state to a fungi-dominated state associated with pathogens. This study provides crucial insights for developing more sustainable farming practices, such as using beneficial bacteria as biofertilizers or improving nutrient management, to combat soil sickness in celery production.

## 1. Introduction

Celery (*Apium graveolens* L.) is a biennial leafy vegetable belonging to the Apiaceae family, renowned for its abundant nutritional and medicinal properties, and is extensively cultivated on a global scale [1,2]. In the context of protected cultivation, particularly within greenhouse systems, celery is frequently cultivated in successive cycles to optimize land use efficiency and satisfy year-round market demand. In conventional greenhouse soil-based production systems, celery can be harvested three to four times annually from the same soil [3]. Although this intensification is economically advantageous, it has raised concerns regarding the long-term sustainability of continuous monoculture practices.

Continuous cropping, defined as the repeated cultivation of the same crop species on the same land without rotation, is a common practice in intensive vegetable production worldwide. However, prolonged monoculture frequently leads to a suite of interconnected problems collectively termed “continuous cropping obstacles” or “soil sickness” [4,5,6]. The main factors underlying continuous cropping obstacles currently include soil nutrient imbalance [7], accumulation of autotoxic allelochemicals [8], proliferation of soilborne pathogens [9], and monoculture-related soil degradation [10]. Consequently, continuous cropping is associated with progressive yield decline, reduced product quality, and increased dependence on agrochemical inputs [11,12]. While continuous cropping-induced soil sickness has been documented across diverse cropping systems—including watermelon [13], cucumber [14], and tomato [15]—the mechanisms vary considerably among crop species and remain incompletely understood for many vegetable crops, including celery.

The rhizosphere, the narrow zone of soil directly influenced by root exudates, serves as the primary interface for plant-microbe interactions and governs plant nutrition, stress tolerance, and disease resistance [16,17]. Rhizosphere microbial communities—often referred to as the plant’s “second genome”—play critical roles in nutrient cycling, phytohormone production, and pathogen suppression [18]. Under continuous cropping, the composition and function of these microbial communities undergo directional shifts in response to sustained selective pressures imposed by repeated root exudation and altered soil conditions [19].

Bacteria and fungi differ fundamentally in their life history strategies, nutrient acquisition mechanisms, and sensitivity to environmental stress [20]. Bacteria typically have higher nutrient demands, faster growth rates, and greater sensitivity to substrate depletion, whereas fungi are generally more tolerant of low-nutrient conditions, can access recalcitrant organic matter via extracellular enzymes, and maintain extensive hyphal networks that buffer against localized resource scarcity [21,22]. These contrasting ecological traits suggest that bacterial and fungal communities may respond diametrically to continuous cropping-induced soil changes. Specifically, under conditions of nutrient depletion—particularly of readily available nitrogen and potassium—bacterial communities would be expected to decline in diversity and abundance, whereas fungal communities might proliferate, potentially including saprotrophic and pathogenic taxa adapted to low-nutrient or stress-altered environments [20,23]. This conceptual framework, while plausible, has not been rigorously tested in celery continuous cropping systems. Previous studies have demonstrated that functional keystone taxa play critical roles in nitrogen transformation in agricultural systems [24,25]. Additionally, microbial community structure has been shown to be closely linked to nitrogen mineralization rates [26]. Furthermore, the decomposition of organic matter and associated microbial community dynamics have been extensively studied in composting systems [27,28,29,30].

For celery, the issue of continuous cropping is particularly relevant. Celery possesses a shallow, fibrous root system with limited lateral spread, making it heavily dependent on the quality of the upper soil layer (0–20 cm) for nutrient and water acquisition [3]. Under continuous monoculture, celery is known to be susceptible to *Fusarium* wilt caused by *Fusarium oxysporum* f. sp. *apii*, a soilborne pathogen that can persist in soil for years and cause devastating yield losses [31,32]. Moreover, celery is often grown in greenhouses where crop rotation is economically disincentivized and technically challenging to implement [33]. However, low short-term profitability, rigidity of the agricultural industrial chain, and insufficient policy support have rendered crop rotation difficult to implement in facility agriculture [34]. Despite these vulnerabilities, the integrated effects of continuous cropping on soil nutrient dynamics and the coordinated responses of bacterial and fungal communities in the celery rhizosphere have not been systematically investigated. In particular, it remains unclear how continuous cropping alters soil physicochemical properties and which specific nutrients are most affected, whether bacterial and fungal communities show opposing diversity and compositional responses as predicted by their contrasting ecological strategies, which specific beneficial or pathogenic taxa are depleted or enriched, and how nutrient-microbe coupling relationships are altered under continuous cropping.

To address these knowledge gaps, we conducted a comparative study of celery rhizosphere soils from continuous cropping (CC, four consecutive cycles) and non-continuous cropping (CK, first planting) systems in a commercial greenhouse in Liaocheng, eastern China. Using high-throughput sequencing (16S rRNA for bacteria, ITS for fungi), comprehensive soil physicochemical analysis, and multivariate statistical approaches (Mantel tests, LEfSe, NMDS), we systematically compared the two systems. We hypothesized that continuous cropping induces significant depletion of labile nutrients (particularly available N and K), leading to opposing diversity responses of bacteria (decrease) versus fungi (increase), accompanied by depletion of beneficial plant growth-promoting rhizobacteria (e.g., *Bacillus*, *Pseudomonas*) and enrichment of the pathogenic genus *Fusarium*, together with altered nutrient-microbe coupling relationships. The findings of this study are expected to provide a mechanistic understanding of continuous cropping obstacles in celery and to inform the development of microbiome-based and nutrient management strategies for sustainable celery production.

## 2. Materials and Methods

### 2.1. Study Site and Soil Sampling

The study site was located in Liaocheng, Shandong Province, eastern China (36°26′ N, 115°58′ E), within the warm temperate monsoon climate zone. The region receives 2463–2742 h of sunlight annually, with a mean annual temperature of 12.8–13.4 °C and annual precipitation of 567–637 mm. The frost-free period lasts approximately 200 days.

The experimental plots were situated in a commercial vegetable greenhouse operated by Liaocheng Chuangjufeng Wanjiang Agricultural Technology Development Co., Ltd. Two treatments were established: continuous cropping (CC) and non-continuous cropping (CK). The CC plots had been cultivated with celery (*Apium graveolens* L. cv. ‘Qianfeng’) for four consecutive cropping cycles (each cycle lasted 80–100 days; approximately 16 months of continuous monoculture). The CK plots represented the first celery planting on adjacent land that had been fallow for one season prior to planting. Both treatments were arranged in a completely randomized design with three plot replicates per treatment (CC, *n* = 3; CK, *n* = 3). Each plot measured approximately 30 m^2^ (5 m × 6 m).

All agronomic practices during the sampled growth cycle were identical between CC and CK plots. A standard fertilization regime was applied: compound fertilizer (N:P_2_O_5_:K_2_O = 15:15:15) at 450 kg·ha^−1^ per cycle as base fertilizer, supplemented with urea (46% N) at 150 kg·ha^−1^ per cycle as topdressing. No fungicides were applied during the experiment or in the previous cycles. Irrigation was applied via drip irrigation (approximately 300 m^3^·ha^−1^ per cycle), and tillage was performed uniformly across all plots.

At the leaf cluster growth stage (plant height approximately 30 cm), rhizosphere soil samples were collected. For each plot, five random plants were selected. Root systems with adhering soil were carefully excavated from the 0–20 cm depth using a sterile spade. For each plant, the entire root system was gently shaken to remove loosely attached bulk soil; the soil that remained firmly attached to the root surface was defined as rhizosphere soil. The five subsamples from each plot were pooled to form one composite sample, yielding three biological replicates per treatment. The same composite samples were used for both physicochemical analysis and high-throughput sequencing.

Samples for DNA extraction were immediately frozen in liquid nitrogen and stored at −80 °C. Samples for physicochemical analysis were air-dried, sieved through a 2 mm mesh, and stored at room temperature in sealed containers. All samples were collected on 23 March 2025.

### 2.2. Physicochemical Analysis

Each composite soil sample was analyzed in three technical replicates for all physicochemical parameters. Results are presented as mean ± standard deviation. Total nitrogen (TN): Quantified using sulfuric acid digestion followed by Kjeldahl determination (Kjeltec 8400, FOSS, Hillerød, Denmark). Total phosphorus (TP): Determined using the NaOH fusion method followed by molybdenum-antimony spectrophotometric detection (Shimadzu UV-2600, Shimadzu Corporation, Kyoto, Japan). Total potassium (TK): Measured using NaOH fusion followed by flame photometry (FP640, Shanghai INESA Analytical Instrument Co., Ltd., Shanghai, China). Available potassium (AK): Extracted using 1 M NH_4_OAc (pH 7.0) and quantified by flame photometry. Alkali-hydrolyzable nitrogen (AN): Determined using the alkaline diffusion method (Conway micro-diffusion). The physicochemical parameters above were tested according to Lu (2000) [35]. Available phosphorus (AP): Extracted using 0.5 M NaHCO_3_ (Olsen method) at pH 8.5, followed by molybdenum-antimony colorimetric determination [36]. Soil organic carbon (OC): Measured using the Walkley-Black potassium dichromate oxidation method with external heating [37,38]. Soil pH: Measured in a 1:2.5 (*w*/*v*) soil:water suspension using a calibrated pH meter (Mettler Toledo, Greifensee, Switzerland).

### 2.3. DNA Extraction and PCR Amplification

Total microbial genomic DNA was extracted from 0.5 g of soil (per sample) using the E.Z.N.A.^®^ Soil DNA Kit (Omega Biotek, Norcross, GA, USA) following the manufacturer’s protocol. Extraction blanks (no soil) were processed alongside samples to monitor contamination; no amplification was detected in these blanks. DNA purity was assessed by A_260_/A_280_ ratio (all samples between 1.8 and 2.0) and A_260_/A_230_ ratio (>1.8) using a NanoDrop^®^ ND-2000 spectrophotometer (Thermo Scientific, Waltham, MA, USA). All samples were diluted to 1 ng·μL^−1^ to reduce potential PCR inhibitors. DNA samples were stored at −80 °C.

The hypervariable V3–V4 region of the bacterial 16S rRNA gene was amplified using primers 338F (5′-ACTCCTACGGGAGGCAGCAG-3′) and 806R (5′-GGACTACHVGGGTWTCTAAT-3′) [34]. The internal transcribed spacer (ITS) region of the fungal rRNA operon (ITS1–5.8S–ITS2) was amplified using primers ITS1F (5′-CTTGGTCATTTAGAGGAAGTAA-3′) and ITS2R (5′-GCTGCGTTCTTCATCGATGC-3′). Different PCR conditions were used for the two markers, as confirmed by the sequencing provider: The PCR amplification cycling conditions for 16S rRNA gene was as follows: initial denaturation at 95 °C for 3 min, followed by 29 cycles of denaturation at 95 °C for 30 s, annealing at 53 °C for 30 s, and extension at 72 °C for 45 s, with a final extension at 72 °C for 10 min. The PCR amplification cycling conditions for the ITS region were as follows: initial denaturation at 95 °C for 3 min, followed by 35 cycles of denaturation at 95 °C for 30 s, annealing at 55 °C for 30 s, and extension at 72 °C for 45 s, with a final extension at 72 °C for 10 min. The PCR reaction mixture (20 μL) contained: 4 μL of 5× Fast Pfu buffer, 2 μL of 2.5 mM dNTPs, 0.8 μL of each primer (5 μM), 0.4 μL of Fast Pfu polymerase (TransGen Biotech, Beijing, China), 10 ng of template DNA, and ddH_2_O to volume. For each PCR run, a negative control (no template DNA) and a positive control (standard DNA from Escherichia coli for 16S and Saccharomyces cerevisiae for ITS) were included. No amplification was observed in negative controls. All samples were amplified in triplicate (three independent PCR reactions), and the products were pooled to minimize reaction-to-reaction variation.

PCR products were separated on 2% agarose gels, excised, and purified using the AxyPrep DNA Gel Extraction Kit (Axygen Biosciences, Union City, CA, USA) according to the manufacturer’s instructions, and quantified using a Quantus™ Fluorometer (Promega, Madison, WI, USA).

Library preparation: Barcoded primers containing sample-specific index sequences were used in a two-step PCR amplification protocol. The first PCR amplified the target region (conditions as above). The second PCR (8 cycles) added Illumina sequencing adapters and dual-index barcodes. Libraries were purified using AMPure XP beads (Beckman Coulter, Brea, CA, USA) and quantified using a Qubit 4.0 fluorometer (Thermo Scientific, Waltham, MA, USA).

### 2.4. Illumina Miseq Sequencing

Library quality was assessed on an Agilent 2100 Bioanalyzer (Agilent Technologies, Santa Clara, CA, USA) using a High Sensitivity DNA Kit (Agilent Technologies, Santa Clara, CA, USA). Libraries were normalized to 4 nM, pooled in equimolar amounts, and subjected to paired-end sequencing (2 × 300 bp) on the Illumina MiSeq platform (Illumina, San Diego, CA, USA) using the MiSeq Reagent Kit v3 (600 cycles) (Illumina, San Diego, CA, USA), following the standard protocols of Majorbio Bio-Pharm Technology Co., Ltd. (Shanghai, China). The raw sequencing reads have been deposited in the NCBI Sequence Read Archive (SRA) under BioProject accession number PRJNA1453622.

### 2.5. Bioinformatics Analysis

Quality filtering and merging: Raw FASTQ files were demultiplexed using a custom Perl script. Quality filtering was performed with fastp version 0.19.6 [39] using the following criteria: (i) reads were truncated at any position where the average quality score fell below 20 over a 50 bp sliding window (applied independently to forward and reverse reads); (ii) reads shorter than 50 bp after trimming were discarded; (iii) reads containing ambiguous characters (N) were discarded. Paired-end reads were merged using FLASH version 1.2.7 [40] with a minimum overlap of 10 bp and a maximum mismatch ratio of 0.2.

Separate processing of 16S and ITS: 16S and ITS datasets were processed separately through all bioinformatics steps. For ITS data, an additional step of removing ITS1 and ITS2 flanking regions using ITSx was performed before OTU clustering.

OTU clustering: Merged sequences were clustered into operational taxonomic units (OTUs) at 97% sequence similarity using UPARSE version 7.1 [41,42]. The most abundant sequence in each OTU was selected as the representative sequence. While ASV-based methods (e.g., DADA2) offer higher resolution, we retained the OTU approach to maintain comparability with earlier continuous cropping studies that used OTU-based analysis.

Taxonomic annotation: Taxonomic assignment of bacterial OTUs was performed using the RDP Classifier (confidence threshold 0.7) against the SILVA 138.1 rRNA database (97% similarity). Fungal OTUs were assigned using the UNITE 9.0 database (dynamic thresholds) with the same confidence cutoff.

Rarefaction and diversity analyses: To mitigate the impact of sequencing depth, all samples were rarefied to 51,822 reads for 16S, and 62,560 reads for ITS (based on the minimum library size after quality filtering). Alpha diversity indices (ACE, Chao, Sobs, Shannon, Simpson) and Good’s coverage were calculated using Mothur version 1.30.1 [43] on rarefied OTU tables. This approach yielded an average Good’s coverage of 99%.

### 2.6. Statistical Analysis

Soil microbial community similarity was assessed using non-metric multidimensional scaling (NMDS) based on Bray–Curtis dissimilarity with the vegan package (version 2.5-3) in R. Analysis of similarities (ANOSIM) was performed using the same distance metric with 999 permutations to evaluate treatment differences. Linear discriminant analysis effect size (LEfSe) [44] was used to identify differentially abundant taxa (phylum to genus) between groups, with an LDA score threshold > 2 and *p* < 0.05.

Results are presented as mean ± standard deviation (SD). Significant differences in soil fertility, diversity, and richness indices between CC and CK were identified using one-way ANOVA followed by Duncan’s multiple range test, with significance levels set at *p* < 0.05 and *p* < 0.01. All statistical analyses were conducted using SAS version 9 (SAS Institute Inc., Cary, NC, USA).

## 3. Results

### 3.1. Soil Physicochemical Properties

The comparative analysis of soil physicochemical properties (Table 1) revealed that, compared to CK (control, first celery planting after one season of fallow), CC (four consecutive celery cycles) soils exhibited significantly higher total phosphorus (TP) (2.19 vs. 2.03 g/kg, +8.0%, *p* < 0.01). In contrast, total nitrogen (TN), total potassium (TK), available phosphorus (AP), available potassium (AK), alkali-hydrolyzable nitrogen (AN), and organic carbon (OC) were all significantly lower in CC soils (all *p* < 0.01). The most pronounced depletions were observed for AK (130.7 vs. 433.7 mg/kg, −69.9%) and AN (49.2 vs. 144.3 mg/kg, −65.9%). The combination of elevated TP but reduced AP in CC soils suggests potential phosphorus fixation or microbial immobilization. Soil pH did not differ significantly between treatments (7.82 vs. 7.89, *p* > 0.05).

### 3.2. Sequencing Quality Evaluation

High-throughput sequencing of the 16S rRNA gene amplicon (bacteria) and ITS region (fungi) was performed on the Illumina MiSeq platform (2 × 300 bp). After quality filtering and chimera removal, a total of 52,654 bacterial reads (CC) and 57,721 bacterial reads (CK) were obtained, with an average of 55,188 reads per bacterial sample (range: 51,822–59,979 reads). For fungi, a total of 64,918 reads (CC) and 76,708 reads (CK) were obtained, with an average of 70,813 reads per fungal sample (range: 62,560–81,729 reads). The average read lengths were 418 bp (bacteria) and 232 bp (fungi), consistent with the targeted amplicon sizes. Quality scores were high, with Q20 values exceeding 98% and Q30 values exceeding 94% for all samples, indicating reliable base calling.

Sequence quality filtering was performed using fastp version 0.19.6 with a 10 bp sliding window (average quality threshold Q20). After trimming, paired-end reads were merged using FLASH version 1.2.7 with a minimum overlap of 10 bp. The optimized sequences were clustered into operational taxonomic units (OTUs) at 97% sequence similarity using UPARSE version 7.1. A total of 3282 bacterial OTUs and 4110 fungal OTUs were identified across all samples. Good’s coverage exceeded 98.8% for all bacterial samples and 99.9% for all fungal samples (Table 2), confirming that the sequencing depth adequately captured the majority of microbial diversity.

**Figure 1:**
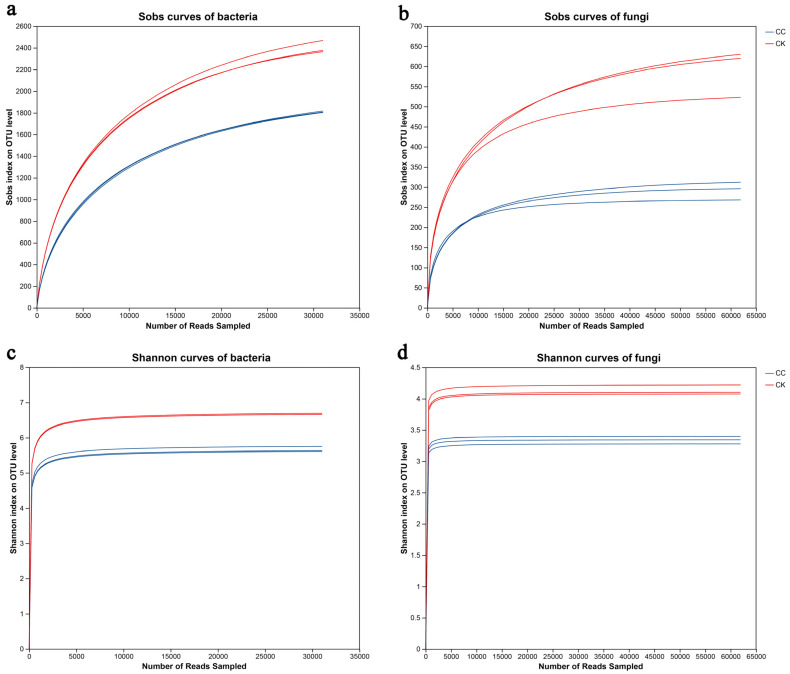
Sobs curves of bacterial (**a**) and fungal (**b**) and Shannon curves of bacterial (**c**) and fungal (**d**) were examined to assess the impact of a 3% dissimilarity cutoff on the identification of uncovered operational taxonomic units (OTUs). The term “CC” denotes the continuous cropping celery rhizosphere soil group, whereas “CK” signifies the control group without continuous cropping plantation.

To evaluate whether sequencing depth was sufficient for downstream analyses, rarefaction curves were generated for both Sobs (observed OTUs) and Shannon diversity indices (Figure 1). The Sobs rarefaction curves for both bacterial and fungal communities did not reach a plateau (Figure 1a,b), indicating that richness-based metrics may still be sensitive to rare taxa and that additional sequencing could potentially recover more OTUs. In contrast, the Shannon diversity rarefaction curves approached a clear plateau for both bacteria and fungi (Figure 1c,d), suggesting that the sequencing depth was sufficient for comparative analyses of α-diversity and community structure between treatments. Based on the minimum library size after quality filtering (bacteria: 51,822 reads; fungi: 62,560 reads), all samples were rarefied to 50,000 reads for bacterial analyses and 60,000 reads for fungal analyses for subsequent diversity and community composition analyses.

### 3.3. α-Diversity and β-Diversity Analysis

Bacterial α-diversity: Continuous cropping significantly reduced bacterial richness and diversity (Table 2). The richness estimators ACE, Chao, and Sobs were significantly lower in CC than in CK (all *p* < 0.01). Specifically, the Sobs index decreased from 2402 ± 56 in CK to 1809 ± 7 in CC (*p* < 0.01). Shannon diversity was lower in CC (5.66 vs. 6.67, *p* < 0.01), while Simpson diversity was higher in CC (0.003 vs. 0.019, *p* < 0.01), indicating reduced evenness and greater dominance of certain taxa in CC soils.

Fungal α-diversity: In striking contrast to bacteria, fungal richness and diversity increased dramatically under continuous cropping (Table 2). ACE, Chao, and Sobs were approximately two-fold higher in CC than in CK (all *p* < 0.01). Shannon diversity was higher in CC (4.13 vs. 3.34, *p* < 0.01), while Simpson diversity was lower in CC (0.042 vs. 0.071, *p* < 0.01), indicating higher evenness and reduced dominance in CC soils.

**Figure 2:**
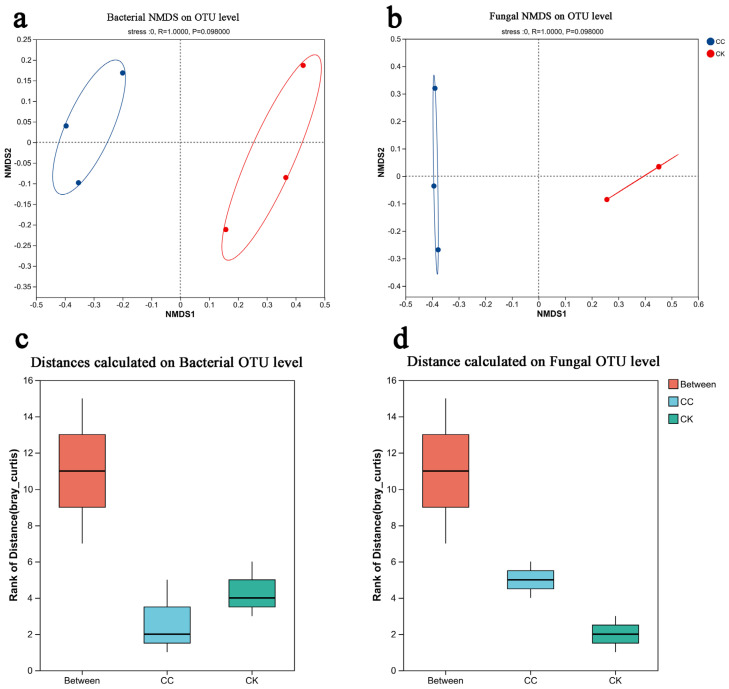
NMDS analysis and ANOSIM analysis of continuous cropping celery and non-continuous cropping cultivation celery rhizosphere soil microbes. The term “CC” denotes the continuous cropping celery rhizosphere soil group, whereas “CK” signifies the control group without continuous cropping plantation. (**a**) NMDS analysis of bacteria on OTU level. (**b**) NMDS analysis of fungi on OTU level. (**c**) ANOSIM analysis of bacteria on OTU level. (**d**) ANOSIM analysis of fungi on OTU level.

β-diversity analysis: NMDS based on Bray–Curtis dissimilarity revealed clear separation between CC and CK communities for both bacteria and fungi (Figure 2a,b). The stress values (bacteria: 0.062; fungi: 0.058) indicated excellent ordination fit. NMDS ordination demonstrated clear separation between CC and CK groups, with inter-group distances substantially exceeding intra-group distances. Replicates within each treatment formed tight clusters, indicating high compositional similarity among biological replicates, while the marked separation between groups reflects fundamental differences in community structure induced by continuous cropping. ANOSIM confirmed that inter-group dissimilarity was significantly greater than intra-group dissimilarity (bacteria: R = 0.889, *p* = 0.001; fungi: R = 0.852, *p* = 0.001; Figure 2c,d), indicating that continuous cropping fundamentally restructured both bacterial and fungal community composition.

### 3.4. Microbial Community Composition and Structure

**Figure 3:**
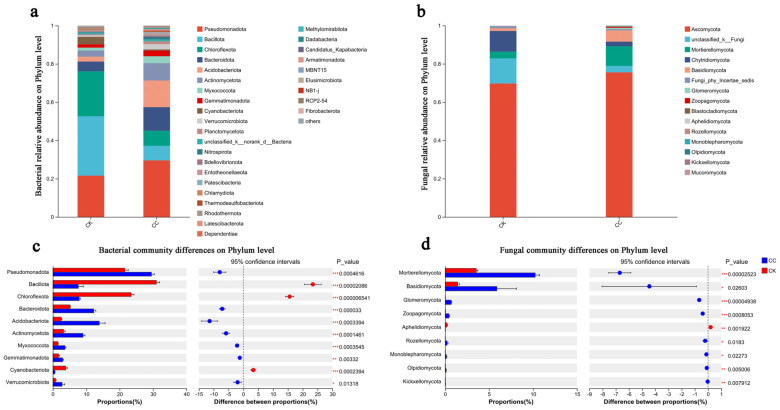
Composition and differences in bacteria and fungi at the phylum level. (**a**) Relative abundances of bacteria at the phylum level. (**b**) Relative abundances of fungi at the phylum level. (**c**) Differences in bacteria at the phylum level. (**d**) Differences in fungi at the phylum level. The term “CC” denotes the continuous cropping celery rhizosphere soil group, whereas “CK” signifies the control group without continuous cropping plantation. Asterisks indicate statistically significant differences: * *p* < 0.05, ** *p* < 0.01, *** *p* < 0.001.

Bacterial phylum-level composition: The bacterial communities of CC and CK shared the same dominant phyla (relative abundance > 5% in either treatment), but their proportions differed substantially (Figure 3a,c). In CC soils, *Pseudomonadota* (Proteobacteria) and Acidobacteriota increased significantly (29.55% vs. 21.57%, *p* < 0.001; 13.92% vs. 2.53%, *p* < 0.001, respectively), while *Bacillota* (Firmicutes), *Chloroflexota*, and *Bacteroidota* decreased significantly (7.64% vs. 31.03%, *p* < 0.001; 7.90% vs. 23.48%, *p* < 0.001; 12.28% vs. 5.13%, *p* < 0.001, respectively). The decline of *Bacillota*, a phylum encompassing many plant growth-promoting and stress-tolerant species, suggests a loss of beneficial functions under continuous cropping. The concurrent rise in *Acidobacteriota*, often associated with oligotrophic conditions and recalcitrant organic matter degradation, aligns with the observed depletion of labile nutrients (AK, AN, OC) and suggests an adaptive shift toward nutrient-efficient bacterial taxa.

Fungal phylum-level composition: Ascomycota dominated both treatments (75.56% in CC, 69.75% in CK; *p* > 0.05). *Mortierellomycota* decreased significantly in CC (3.37% vs. 13.11%, *p* < 0.001), while Basidiomycota increased (5.89% vs. 1.43%, *p* < 0.05). Although *Chytridiomycota* showed lower mean abundance in CC (2.41% vs. 10.78%), high within-group variability resulted in no statistical significance (*p* > 0.05). The decline of *Mortierellomycota*, a phylum known for phosphate solubilization and biocontrol potential, may compromise beneficial fungal functions in CC soils.

**Figure 4:**
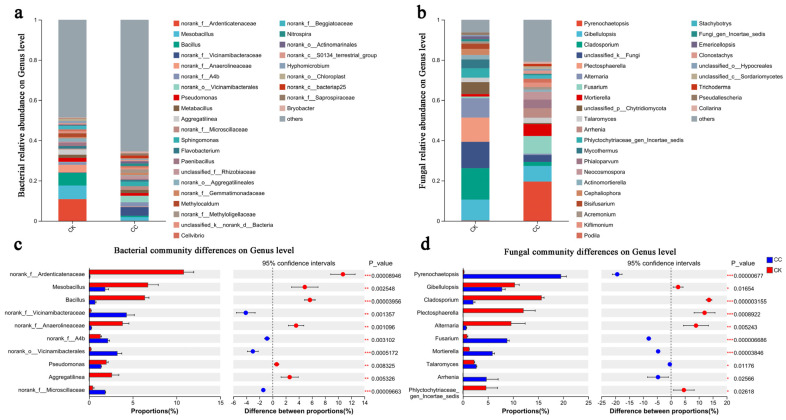
Composition and differences in bacteria and fungi at the genus level. (**a**) Relative abundances of bacteria at the genus level. (**b**) Relative abundances of fungi at the genus level. (**c**) Differences in bacteria at the genus level. (**d**) Differences in fungi at the genus level. The term “CC” denotes the continuous cropping celery rhizosphere soil group, whereas “CK” signifies the control group without continuous cropping plantation. Asterisks indicate statistically significant differences: * *p* < 0.05, ** *p* < 0.01, *** *p* < 0.001.

Bacterial genus-level composition: The dominant bacterial genera (mean relative abundance > 1% in either treatment) included *Mesobacillus*, *Bacillus*, *Pseudomonas*, and *Metabacillus* (Figure 4a,c). *Bacillus* declined dramatically from 6.34% in CK to 0.68% in CC (*p* < 0.001, −89.3%). *Mesobacillus* decreased from 6.72% to 1.83% (*p* < 0.01, −72.8%). *Pseudomonas* decreased from 1.98% to 1.37% (*p* < 0.01, −30.8%). In contrast, *Metabacillus* showed no significant difference (1.59% vs. 1.25%, *p* > 0.05). The severe depletion of *Bacillus* and *Mesobacillus*, both well-documented plant growth-promoting rhizobacteria (PGPR), likely contributes to the loss of soil suppressiveness.

Fungal genus-level composition: The dominant fungal genera differed markedly between treatments (Figure 4b,d). *Pyrenochaetopsis* increased from 2.22% in CK to 19.54% in CC (*p* < 0.001). *Fusarium*—a genus containing the celery wilt pathogen *F. oxysporum* f. sp. apii—increased 10.9-fold from 0.81% in CK to 8.81% in CC (*p* < 0.001). *Cladosporium* decreased from 15.67% to 2.04% (*p* < 0.001). *Alternaria* decreased from 9.61% to 0.66% (*p* < 0.01). *Gibellulopsis* decreased from 10.32% to 7.78% (*p* < 0.05). The dramatic enrichment of *Fusarium* in CC soils, coupled with the depletion of *Bacillus*, indicates a fundamental shift in the balance between beneficial and pathogenic microbial communities. It is important to note that increased fungal diversity under continuous cropping is not necessarily a favorable indicator; it may reflect the accumulation of potentially pathogenic or stress-tolerant taxa, as suggested by the enrichment of *Fusarium* in CC soils.

**Figure 5:**
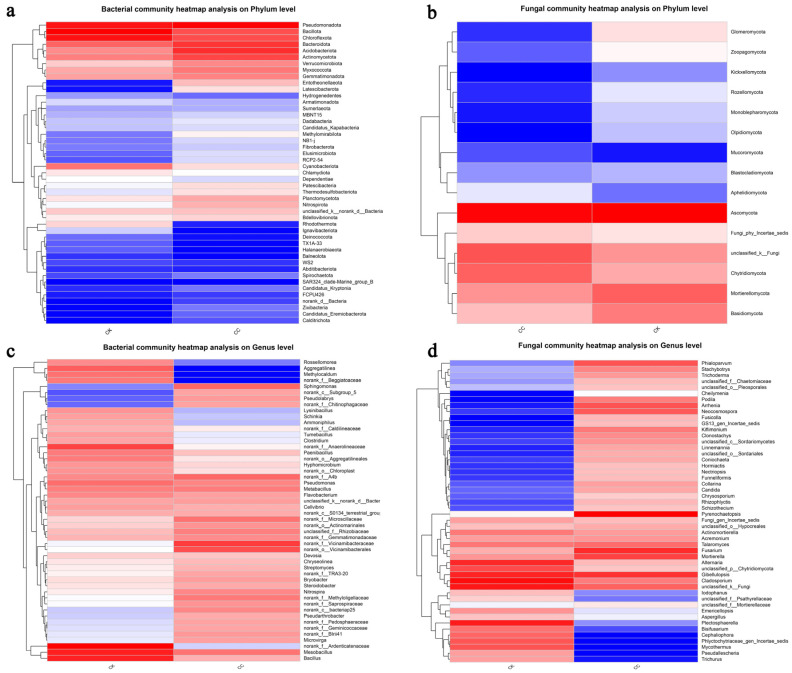
Hierarchical clustering of bacterial and fungal distributions. (**a**) Relative abundances of bacteria at the phylum level. (**b**) Relative abundances of fungi at the phylum level. (**c**) Relative abundances of bacteria at the genus level. (**d**) Relative abundances of fungi at the genus level. The term “CC” denotes the continuous cropping celery rhizosphere soil group, whereas “CK” signifies the control group without continuous cropping plantation.

The results of the hierarchical clustering of bacterial and fungal distributions, as shown in the heatmap provided, confirm the community bar plot. Specifically, they confirmed that *Pseudomonadota*, *Bacillota*, *Chloroflexota*, *Bacteroidota*, *Acidobacteriota*, and *Actinomycetota* are the main components with higher proportions in the bacterial phylum in both groups. Moreover, the CK group has very little content of *Entotheonellaeota* and *Latescibacterota*, and the CC group has very little content of Hydrogenedentes and Dependentiae (Figure 5a). The genera of *Bacillus*, *Mesobacillus*, *Pseudomonas*, *Metabacillus*, and *Flavobacterium* are the predominant genera, with higher relative abundance in the bacterial genera in both groups. On the other hand, the genera of *Aggregatilinea* and *Methylocaldum* showed particularly low relative content in the CC group, and the genera of *Sphingomonas* and *Pseudolabrys* showed particularly low relative content in the CK group (Figure 5c).

The Ascomycota, Chytridiomycota, Mortierellomycota, and Basidiomycota phyla are the main components with higher proportions in the fungal phylum in both groups, which is consistent with the community bar plot. The fungal phylum of *Mucoromycota* and *Aphelidiomycota* showed a particularly low relative content in the CC group. Moreover, the CK group has very little content of *Glomeromycota* and *Rozellomycota* (Figure 5b). The genera of *Pyrenochaetopsis*, *Gibellulopsis*, *Cladosporium*, and *Fusarium* are the predominant genera with higher relative abundance in the fungal genera in both groups. In addition, the genera of *Mycothermus*, *Pseudallescheria*, and *Trichurus* showed relatively low content in the CC group, and the genera of Arrhenia, Podila, and Neocosmospora showed particularly low relative content in the CK group (Figure 5d).

**Figure 6:**
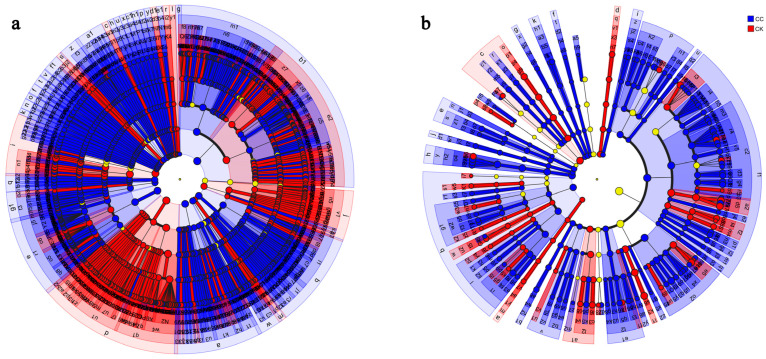
Discriminant analysis of multi-level species differences through LEfSe analysis. (**a**) Differences in bacterial multi-level species in the CC and CK groups. (**b**) Differences in fungal multi-level species in the CC and CK groups. Different-colored nodes represent microbial communities that are significantly enriched in their corresponding groups and have a significant impact on intergroup differences.

LEfSe analysis identified biomarkers specific to each treatment (Figure 6). CK soils were enriched in beneficial bacterial genera (*Bacillus*, *Mesobacillus*, *Paenibacillus*) and fungal genera (*Cladosporium*, *Alternaria*, *Gibellulopsis*). CC soils were characterized by bacterial genera (*Sphingomonas*, *Nitrospira*, *Pseudolabrys*) and fungal genera (*Fusarium*, *Arrhenia*, *Mortierella*, *Neocosmospora*).

### 3.5. Effects of Environmental Factors on Microbial Communities

**Figure 7:**
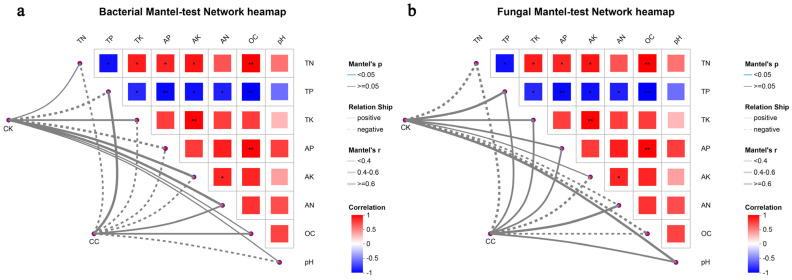
Mantel-test network heatmap. (**a**) The correlation between environmental factors and bacterial community structure. (**b**) The correlation between environmental factors and fungal community structure. The term “CC” denotes the continuous cropping celery rhizosphere soil group, whereas “CK” signifies the control group without continuous cropping plantation. Asterisks indicate statistically significant differences: * *p* < 0.05, ** *p* < 0.01.

Mantel tests were used to explore correlations between soil physicochemical properties and microbial community structure (Figure 7). For bacterial communities in CC soils, significant correlations were observed with TP (r = 0.866, *p* = 0.016), AN (r = 0.945, *p* = 0.008), and OC (r = 0.912, *p* = 0.012). For fungal communities in CC soils, significant correlations were found with TP (r = 0.866, *p* = 0.016), TK (r = 0.680, *p* = 0.048), AP (r = 0.714, *p* = 0.040), and AN (r = 0.714, *p* = 0.038). In CK soils, bacterial communities correlated with TK, AK, and AN, while fungal communities correlated with TP, TK, and pH.

No individual nutrient variable correlated significantly with bacterial or fungal community structure in either CC or CK soils (all *p* > 0.05), likely due to limited statistical power from the small number of replicates (*n* = 3 per group). Nevertheless, the opposite direction of Mantel r values for TP between bacteria (−0.866 in CC) and fungi (+0.866 in CC) is descriptively consistent with the observed phosphorus accumulation in CC soils, suggesting that TP may act as an environmental filter differentially shaping bacterial and fungal communities.

## 4. Discussion

### 4.1. Continuous Cropping Was Associated with Soil Nutrient Depletion with Differential Effects on N, K, and P

The soil physicochemical analysis revealed substantial nutrient alterations in CC compared to CK (Table 1). The patterns most prominently associated with continuous cropping were the marked depletion of AK (−69.9%) and AN (−65.9%), followed by significant reductions in TN, OC, TK, and AP. In contrast, TP showed a modest but statistically significant increase (+8.0%). This pattern of relative N and K depletion with P accumulation is characteristic of intensive continuous cropping systems, where repeated crop uptake of N and K may exceed nutrient replenishment, while P fixation or microbial immobilization could lead to its relative accumulation. Studies in greenhouse cucumber systems have similarly reported decoupling of phosphorus from nitrogen and potassium under continuous cropping, where leaf P concentration increased while N and K remained unchanged, suggesting that imbalanced nutrient availability may alter plant elemental stoichiometry [45].

Several important caveats must be acknowledged. First, our study design (comparison of CC and CK without direct experimental manipulation of nutrient inputs) precludes causal conclusions. Therefore, we describe associations rather than causal effects. Second, we did not directly measure P fractions, phosphatase activity, or phosphate-solubilizing microorganisms. Thus, the interpretation of P fixation or immobilization remains a hypothesis that requires direct validation in future studies. Third, the magnitude of TP increase was modest (8.0%), and its biological significance should not be overstated. Fourth, while nutrient depletion is the most pronounced physicochemical change measured here, we acknowledge that continuous cropping alters multiple soil properties simultaneously. Other factors—including root exudate accumulation, changes in residue quality, and pathogen pressure—may also contribute to microbial community restructuring and are discussed in Section 4.3 and Section 4.6.

This nutrient imbalance pattern has been observed in other continuous cropping systems, although comparisons must be made cautiously. In a five-year watermelon study, researchers observed decreases in SOM, AN, AP, and AK accompanied by a pH decline [13]. A ginseng study showed that soil sterilization treatments significantly affected AK and AN contents [46]. In contrast, an industrial hemp study revealed increasing trends in AN and AK despite continuous cropping [47]. These discrepancies likely reflect differences in crop species, cultivation practices, fertilization regimes, root exudate profiles, and initial soil conditions. For celery, a leafy vegetable with high N and K demands, nutrient depletion may be particularly pronounced.

Notably, soil pH did not differ significantly between CC and CK (7.82 vs. 7.89), contrasting with some previous reports of acidification under continuous cropping [13]. This difference may reflect the initial buffering capacity of the soil, fertilization practices, or crop-specific effects on rhizosphere pH. The stable pH in our study suggests that the observed microbial shifts were not confounded by pH changes, simplifying interpretation.

### 4.2. Opposing Diversity Responses of Bacterial and Fungal Communities to Continuous Cropping

The α-diversity analysis revealed markedly divergent responses: bacterial richness and diversity decreased in CC, while fungal richness and diversity increased approximately two-fold (Table 2). These opposing trends suggest that continuous cropping imposes fundamentally different selective pressures on these two microbial kingdoms.

One plausible explanation is that bacteria and fungi differ in their resource acquisition strategies and stress tolerance. Many bacteria have higher nutrient demands and faster turnover rates compared to fungi, although considerable variation exists within each kingdom, and many bacterial groups are well adapted to oligotrophic conditions. Fungi, generally more tolerant of low-nutrient conditions, can access recalcitrant organic matter via extracellular enzymes and maintain extensive hyphal networks that buffer against localized resource scarcity [20,21,22]. Under the nutrient-depleted conditions observed in CC soils (particularly severe AK and AN reductions), bacterial communities may be disadvantaged, whereas fungi may gain a competitive advantage.

However, several alternative explanations must also be considered. The increase in fungal diversity under continuous cropping could also result from the accumulation of plant debris, changes in root exudate composition, the accumulation of host-specific phytopathogens, or shifts in plant microniche structure. Moreover, increased fungal diversity is not necessarily a favorable indicator; it may reflect the accumulation of potentially pathogenic or stress-tolerant taxa, as suggested by the enrichment of *Fusarium* in CC soils (Section 3.4). Importantly, our data do not directly demonstrate causation, and the interpretations above remain plausible hypotheses requiring experimental validation.

The opposing diversity responses observed in our study align with findings across multiple cropping systems. A recent comparative study on medicinal plants revealed that continuous cropping-sensitive species (*Panax* spp.) aggressively remodeled their rhizosphere, assembling fungal-dominated microbiomes enriched with pathogenic *Nectriaceae*, whereas a resilient species (*Achyranthes bidentata*) maintained bacterial-dominated communities with enriched beneficial *Actinobacteria* [48]. This supports the view that plant ecological strategies may determine susceptibility to continuous cropping obstacles. Similar patterns have been observed in *Erigeron breviscapus* continuous cropping, where increasing planting years led to decreased bacterial diversity and increased fungal dominance, accompanied by the accumulation of soil-borne pathogens [49].

The β-diversity analysis further substantiated the divergence between CC and CK groups. NMDS ordination and ANOSIM confirmed that continuous cropping fundamentally restructures both bacterial and fungal community composition, a finding consistent with previous reports in other continuous cropping systems [23,50,51].

### 4.3. Phylum-Level Shifts Were Associated with Continuous Cropping

At the phylum level (Figure 3), continuous cropping was associated with dramatic compositional shifts. Bacterial communities in CK soils were dominated by *Bacillota* (31.03%) and *Chloroflexota* (23.48%), whereas CC soils showed marked reductions in these phyla (7.64% and 7.90%, respectively) and corresponding increases in *Pseudomonadota* (from 21.57% to 29.55%), *Bacteroidota* (from 5.13% to 12.28%), and *Acidobacteriota* (from 2.53% to 13.92%). Importantly, all abundance data are relative; an increase in the relative abundance of one phylum does not necessarily reflect absolute accumulation and may instead result from the decline of other groups.

The decline of *Bacillota*, a phylum containing many plant growth-promoting and stress-tolerant species, may suggest a loss of beneficial functions under continuous cropping, although not all members of this phylum are beneficial, and functional confirmation is required. This pattern is descriptively consistent with findings from rice-crayfish co-cropping systems, where long-term over-utilization reduced Firmicutes abundance [52], although this comparison is illustrative rather than confirmatory, given the ecological differences between systems.

The concurrent rise in *Acidobacteriota*, often associated with oligotrophic conditions and recalcitrant organic matter degradation, aligns with the observed depletion of labile nutrients (AK, AN, OC) and may suggest an adaptive shift toward nutrient-efficient bacterial taxa [33,53]. The increase in *Bacteroidota*, a phylum often associated with the degradation of readily degradable organic substrates, may reflect changes in soil organic matter quality under continuous cropping. The adaptation of specific bacterial taxa to carbon substrate availability has been documented in various decomposition systems [54].

The enrichment of *Pseudomonadota* in CC soils (from 21.57% to 29.55%) is descriptively consistent with recent insights into the ecological roles of this phylum. Studies have suggested that *Pseudomonadota* can serve as “bridge” taxa linking microbial communities with higher trophic-level consumers, potentially enhancing pathogen suppression [55,56]. A study published in The ISME Journal revealed that *Pseudomonadota* can enhance pathogen suppression through cross-trophic interactions with bacterivorous nematodes [S7], extending our understanding of how phylum-level shifts may translate into functional changes in soil food webs. However, our study did not analyze cross-trophic interactions, co-occurrence networks, or pathogen suppression functions. Therefore, this interpretation remains speculative and requires functional validation.

Linking *Mortierellomycota* decline to soil phosphorus dynamics: For fungal communities, CC was associated with a non-significant increase in Ascomycota (from 69.75% to 75.56%; * *p* * > 0.05) and a significant reduction in *Mortierellomycota* (from 13.11% to 3.37%, * *p* * < 0.001), while Basidiomycota increased significantly (from 1.43% to 5.89%, * *p* * < 0.05). The decline of Mortierellomycota is particularly noteworthy in light of the soil P changes reported in Section 4.1—specifically, the significant decrease in AP (−14.1%) coupled with a modest increase in TP (+8.0%). *Mortierellomycota* is a phylum widely recognized for containing phosphate-solubilizing taxa that enhance P availability through the secretion of organic acids and phosphatases [21,22]. We therefore hypothesize that the marked decline of this phylum under continuous cropping may contribute to the observed reduction in AP, potentially by reducing the abundance and activity of phosphate-solubilizing microorganisms. This hypothesis is further supported by the LEfSe analysis, which identified the genus *Mortierella* (within *Mortierellomycota*) as a specific biomarker of CC soils—suggesting that, despite overall phylum decline, certain members may persist under continuous cropping, warranting species-level investigation.

However, several critical caveats must be emphasized. First, not all members of Mortierellomycota possess phosphate-solubilizing capabilities; functional traits vary considerably among species and strains within the phylum. Second, our abundance data are relative; a decline in relative abundance does not necessarily reflect an absolute decrease in biomass or activity of phosphate-solubilizing taxa. Third, the modest TP increase (8.0%) could reflect P fixation or microbial immobilization, as noted in Section 4.1, and the decline of *Mortierellomycota* may be one of several contributing factors rather than the sole driver. Therefore, the proposed link between *Mortierellomycota* decline and reduced AP remains a testable hypothesis that requires experimental validation through approaches such as: (i) isolation and functional characterization of *Mortierellomycota* strains from CK and CC soils; (ii) direct measurement of phosphatase activity and organic acid production; (iii) P fractionation to distinguish between fixed, organic, and available P pools; and (iv) metatranscriptomic analysis of phosphate solubilization gene expression.

The ecological significance of the Basidiomycota increase remains unclear but may relate to the processing of more stable organic compounds under nutrient-depleted conditions. This pattern aligns with long-term studies on semi-arid farmland, which demonstrated that agricultural management practices significantly alter fungal community structure, with *Mortierellomycota* abundance being particularly sensitive to changes in soil moisture and organic matter decomposition rates [57].

The effects of cropping systems on microbial community structure have also been demonstrated in tobacco, where crop rotation increased Acidobacteria abundance by up to 79% compared to continuous cropping, while continuous cropping enriched Chloroflexi and *Proteobacteria* [58]. Similarly, in Ganoderma lucidum, continuous cropping, bacterial communities shifted significantly with notable changes in *Proteobacteria*, Firmicutes, and *Acidobacteria*, and key *Bacillus* species were identified as strong antagonists against pathogens [50]. This study provides an integrative framework linking species-level bacterial dynamics to continuous cropping barriers in medicinal fungi.

### 4.4. Beyond Nutrient Depletion: Alternative Drivers of Phylum-Level Shifts

While nutrient depletion (particularly AK and AN) is the most directly measured soil physicochemical change in our study and therefore serves as a primary interpretative framework, we acknowledge that continuous cropping alters multiple soil properties and processes simultaneously. Several other factors, which were not directly measured in this study, could independently or synergistically drive the observed phylum-level compositional shifts:

Accumulation of root exudates and allelochemicals: Continuous cropping of the same plant species leads to the accumulation of species-specific root exudates and autotoxic compounds, which can directly inhibit or stimulate particular microbial taxa regardless of nutrient status [8,21]. For example, phenolic acids exuded by celery roots may selectively suppress *Bacillota* while promoting certain *Pseudomonadota*. The mechanistic link between root exudates and beneficial microbe recruitment has been recently elucidated in cucumber Fusarium wilt systems, where resistant cultivars exhibited distinct root exudate profiles enriched in defensive compounds that correlated with the enrichment of beneficial bacteria [14]. Peanut root exudates have also been shown to directly modify soil microbial communities without mediation by nutrient changes [59].

Changes in plant residue quantity and quality: Four consecutive cropping cycles alter the quantity, chemical composition, and decomposition dynamics of plant residues returned to the soil, which in turn shape microbial community structure by favoring taxa adapted to specific carbon substrates [52]. As demonstrated in composting studies, the quantity, chemical composition, and decomposition dynamics of organic residues strongly influence microbial community assembly, favoring taxa adapted to specific carbon substrates [60,61,62].

Phytosanitary pressure (pathogen accumulation): The dramatic enrichment of Fusarium (Section 3.4) indicates that host-specific pathogen accumulation is an important phenomenon in this system. Pathogen establishment can trigger cascade effects on the resident microbial community through antagonistic or competitive interactions, potentially contributing to the decline of *Bacillota* and the rise in *Acidobacteriota* independently of nutrient depletion. As reviewed by Raaijmakers and Mazzola, soil microbial communities can develop “soil immune responses” that suppress or enhance pathogens through complex community-level interactions [63].

Fertilization and water regime: Although fertilization and irrigation were identical across CC and CK plots during the sampled cycle, the legacy effects of four prior cycles of fertilization may have altered soil properties (e.g., salt accumulation, changes in organic matter quality) not captured by our measured parameters. Similarly, long-term continuous cropping can modify soil water retention and aggregate stability, indirectly affecting microbial habitat structure.

Importantly, our observational study design cannot disentangle these co-varying factors. The CC and CK treatments differ in cropping history, which affects multiple soil properties and processes simultaneously. Therefore, while nutrient depletion is the most directly measured change and offers a parsimonious interpretation, the phylum-level shifts reported here should be viewed as associations with continuous cropping rather than conclusive evidence of causation by nutrient depletion alone. Future studies employing controlled experimental designs (e.g., reciprocal soil transplants, root exudate addition experiments, or long-term factorial trials manipulating nutrients and crop rotation independently) are needed to isolate the specific drivers of microbial community restructuring.

### 4.5. Genus-Level Shifts Involving Bacillus, Mesobacillus, and Fusarium

At the genus level (Figure 4 and Figure 5), the most striking changes were observed for bacterial genera that include many strains reported as plant growth-promoting rhizobacteria (PGPR) or biocontrol agents. The relative abundance of *Bacillus* decreased dramatically from 6.34% in CK to 0.68% in CC (*p* < 0.001, −89.3%), and *Mesobacillus* declined from 6.72% to 1.83% (*p* < 0.01, −72.8%). These genera include many well-documented PGPR strains involved in nutrient cycling, phytohormone production, and pathogen suppression. Their severe depletion under continuous cropping may contribute to the loss of soil suppressiveness, although this interpretation requires confirmation through bioassays, plant disease data, or functional markers. Importantly, not all members of *Bacillus* or *Mesobacillus* are beneficial; the genus-level assessment is a simplification that should be refined with species-level identification. Furthermore, our abundance data are relative; a decline in relative abundance does not necessarily reflect an absolute decrease in biomass or activity of these taxa.

This pattern is descriptively consistent with recent findings in *Salvia miltiorrhiza* continuous cropping systems, where planted soils exhibited lower abundances of beneficial bacteria, including *Bacillus*, compared to uncultivated controls [64]. However, such comparisons are illustrative; the systems differ in crop, agricultural background, and soil conditions.

Conversely, the fungal genus *Fusarium*—which includes many plant pathogens—exhibited a dramatic 10.9-fold increase in CC soils (from 0.81% to 8.81%, *p* < 0.001). Additionally, *Pyrenochaetopsis* increased from 2.22% to 19.54% (*p* < 0.001), while *Gibellulopsis* decreased from 10.32% to 7.78% (*p* < 0.05). LEfSe analysis confirmed that *Fusarium*, *Arrhenia*, *Mortierella*, and *Neocosmospora* were specific biomarkers of the CC group, whereas *Cladosporium*, *Alternaria*, *Mycothermus*, and *Gibellulopsis* were enriched in CK. The inclusion of *Mortierella* as a CC biomarker despite the overall decline of Mortierellomycota (Section 4.3) may reflect differential responses at the genus level within a declining phylum—i.e., while the phylum as a whole decreased, the relative abundance of *Mortierella* within the surviving community may have increased, or its persistence made it a statistically significant marker. The inclusion of *Neocosmospora* among CC biomarkers is notable, as this genus is taxonomically close to *Fusarium* and has been associated with phytopathogenic complexes; its enrichment may further indicate shifts toward potentially pathogenic fungal communities under continuous cropping.

*Fusarium oxysporum* f. sp. *Apii* is well documented as the primary causal agent of Fusarium wilt in celery [31]. The 10.9-fold enrichment of the genus *Fusarium* in CC soils suggests that continuous cropping promotes the proliferation of this genus, which includes known pathogens. This finding aligns with a growing body of literature documenting *Fusarium* enrichment under continuous cropping. In watermelon continuous cropping systems, *Fusarium oxysporum* f. sp. *Niveum* (FON) is the primary causal agent of Fusarium wilt, and its accumulation in soil is strongly correlated with disease incidence [32]. However, the genus Fusarium is taxonomically and ecologically heterogeneous; not all members are pathogenic to celery. Similarly, while *Fusarium oxysporum* f. sp. *Niveum* is relevant to watermelon, it serves only as a general reminder that *Fusarium* species can be pathogenic, not as direct evidence for celery. Without species-level identification (e.g., distinguishing *F. oxysporum* from non-pathogenic *Fusarium* species) or pathogenicity testing, the link to disease risk remains a hypothesis. These shifts are consistent with a hypothesis that continuous cropping alters the balance between putatively beneficial and potentially pathogenic taxa, but functional validation is required.

Caution on genus-level functional inference: PGPR is a strain-level functional trait, not a genus-level taxonomic category. Not all members of Bacillus or Mesobacillus are beneficial, and Fusarium includes many non-pathogenic species as well as strains with biocontrol or plant growth-promoting properties. A binary scheme of “beneficial vs. pathogenic” genera oversimplifies real community ecology. Therefore, our interpretations should be understood as patterns consistent with hypotheses about genera that include many functionally important strains, not as confirmed functional assignments. Species- or strain-level resolution (e.g., amplicon sequence variants, whole-genome sequencing, or cultivation) is required to confirm the functional status of specific taxa.

LEfSe analysis identified CC-specific bacterial genera including *Sphingomonas*, *Nitrospira*, *Pseudolabrys*, *Microvirga*, *Priestia*, and *Hassallia*, while CK-specific genera included *Bacillus*, *Mesobacillus*, *Aggregatilinea*, *Methylocaldum*, *Rossellomorea*, and *Paenibacillus*. The interpretation that CK-specific genera are primarily beneficial and CC-specific genera are stress-tolerant is plausible but simplified; at the genus level, confident assignment of ecological function is not always possible. For example, *Sphingomonas* and *Microvirga* can include strains with plant growth-promoting properties. Brief ecological notes on other significant genera: *Pyrenochaetopsis* (enriched in CC) includes species known to colonize stressed or senescing plant tissues; *Nitrospira* (CC biomarker) is a chemolithoautotrophic nitrite-oxidizing bacterium, and its increase may reflect altered nitrogen cycling dynamics; *Paenibacillus* (CK biomarker) includes many PGPR strains, and its depletion aligns with the loss of beneficial functions.

The severe depletion of *Bacillus* in our CC soils (89% decrease) is consistent with multiple recent reports. Ma et al. (2024) demonstrated that in *Lonicera japonica*, continuous cropping, beneficial bacteria including *Bacillus* and *Nitrosospira* were gradually depleted with increasing cropping years, and three *Bacillus* strains isolated from non-continuous cropping soils showed strong antagonism against *Fusarium oxysporum* [65]. Furthermore, a recent ecology-guided study constructed an eight-member *Bacillus* synthetic community (SynCom) from rice-duckweed agroecosystems, revealing that individual strains specialize in auxin production, siderophore-mediated iron mobilization, or lipopeptide/polyketide-based antagonism—functions that collectively promote plant growth and suppress fungal pathogens [66].

### 4.6. Associations Between Environmental Factors and Microbial Communities

Summary of associations: Based on the Mantel test results, the following soil physicochemical properties showed statistically significant correlations with microbial community structure (all *p* < 0.05): Bacterial communities in CC soils: TP, AN, and OC; Fungal communities in CC soils: TP, TK, AP, and AN; Bacterial communities in CK soils: TK, AK, and AN; Fungal communities in CK soils: TP, TK, and pH.

Variables that were not statistically significant in individual tests included AK for CC bacterial communities (*p* > 0.05) and several others. Therefore, AK is not presented as a confirmed modulator in the CC system based on our data.

Several important limitations must be acknowledged. First, no individual nutrient variable correlated significantly with bacterial or fungal community structure in either CC or CK soils when considering all variables simultaneously (global tests: bacteria CC r = 0.554, *p* = 0.094; fungi CC r = 0.721, *p* = 0.056), likely due to limited statistical power from the small number of replicates (*n* = 3 per group). Therefore, we do not claim any of these correlations as confirmed drivers; rather, they point to potentially strong associations that merit investigation with larger sample sizes.

Second, the Mantel test has well-recognized limitations: it has limited power with small sample sizes, and its sensitivity to data structure can produce high r values even when *p*-values are not significant. The observed r = 1.000 for AN vs. bacteria, while descriptively striking, should be interpreted with caution given the non-significant global test and small sample size.

Third, the Mantel test demonstrates associations between distance matrices but does not establish causation. Therefore, we describe associations rather than causal effects. The phrase “environmental factors driving microbial community differentiation” has been revised to “factors associated with community variation”.

Fourth, associations between soil properties and microbial communities may be partially mediated by unmeasured variables, including root exudates, moisture, microzonation, or seasonal effects. These factors were not controlled for in our study design.

The opposite direction of Mantel r values for TP between bacteria (−0.866 in CC) and fungi (+0.866 in CC) is descriptively consistent with the observed phosphorus accumulation in CC soils. This pattern suggests that TP may act as an environmental factor differentially associated with bacterial and fungal community composition. For fungal communities, both TP and AP showed significant correlations; from a biological perspective, available phosphorus (AP) is usually closer to actual microbial responses than total phosphorus, and this should be considered in future mechanistic studies. Similarly, TK (total potassium) showed a significant correlation with fungal communities in CC soils, indicating that potassium dynamics—not only nitrogen and phosphorus—may be relevant to fungal community restructuring under continuous cropping. However, we caution that the magnitude of TP increase was modest (8.0%), and its biological significance should not be exaggerated. Direct measurements of P fractions, phosphatase activity, or phosphate-solubilizing microorganisms would be needed to confirm mechanistic interpretations.

The Mantel test results from our study are broadly consistent with findings from other continuous cropping systems, although comparisons must be made cautiously given differences in study design and analytical depth. In a 21-year watermelon continuous cropping study, structural equation models revealed that variations in soil pH, nutrients, salinity, and moisture content jointly explained 73% and 64% of the variation in bacterial and fungal compositions, respectively; Mantel tests further identified soil moisture and pH as key drivers (Mantel R = 0.74 and 0.54, both *p* < 0.01) [S6]. Our study does not have a comparable depth of analysis, and this comparison is intended only as an illustrative reference, not as direct equivalence.

Similarly, our identification of AN and TP as potentially important correlates of microbial community restructuring aligns with the emerging consensus that continuous cropping obstacles are associated with persistent plant–soil feedbacks arising from rhizosphere microecological reorganization, rather than by bulk soil nutrient depletion alone [50].

### 4.7. A Conceptual Model of Continuous Cropping-Associated Microbiome Shifts

Integrating all findings, we propose a working conceptual model of continuous cropping-associated microbiome shifts in the celery rhizosphere. Based on the observed associations, continuous cropping was associated with (i) severe depletion of available N and K, accompanied by modest P accumulation; (ii) reduced bacterial diversity and richness, with marked declines in genera that include many PGPR strains (e.g., *Bacillus*, *Mesobacillus*); (iii) increased fungal diversity and richness, with dramatic enrichment of the genus *Fusarium* (which *includes* many plant pathogens); (iv) phylum-level shifts from Bacillota/Chloroflexota-dominated to Pseudomonadota/Bacteroidota/Acidobacteriota-dominated bacterial communities; and (v) altered nutrient-microbe associations, with TP, AN, and OC emerging as potentially important correlates for bacterial communities, and TP, TK, AP, and AN for fungal communities.

This model is presented as a working hypothesis, not an established mechanism. Our data are correlational, and causal inferences cannot be drawn from the current study design. The model should be tested experimentally using approaches such as inoculation trials, soil sterilization-transfer experiments, functional bioassays, or absolute quantification methods (e.g., qPCR). Strictly speaking, our data reflect differences in relative abundance and diversity, not absolute suppression or proliferation. Confirmation would require quantitative methods such as qPCR or absolute quantification sequencing.

Caution on genus-level functional inference within the model: The model includes observations about genera that include many beneficial or pathogenic strains. However, PGPR is a strain-level functional trait, not a genus-level taxonomic category. Not all members of *Bacillus* or *Mesobacillus* are beneficial, and *Fusarium* includes many non-pathogenic species as well as strains with biocontrol or plant growth-promoting properties. The binary scheme of “beneficial vs. pathogenic” genera oversimplifies real community ecology. Therefore, our interpretations should be understood as patterns consistent with hypotheses about genera that include many functionally important strains, not as confirmed functional assignments. Species- or strain-level resolution is required to confirm the functional status of specific taxa.

Alternative explanations for the observed patterns must also be considered. As discussed in Section 4.4, changes in root exudate composition could directly affect microbial recruitment without mediation by soil nutrients. Accumulation of autotoxic allelochemicals, shifts in soil physical properties, or changes in water regime could independently influence microbial communities. Pathogen accumulation may not be a consequence of general “dysbiosis” but rather the result of specific host selection and agricultural conditions. Community changes may reflect not only stress and degradation but also a transition to a different stable state of the soil system. Without at least a brief acknowledgment of such alternatives, the model would be overly simplified.

The pattern of reduced bacterial diversity and increased fungal diversity, coupled with the enrichment of *Fusarium*, is consistent with the hypothesis that continuous cropping alters the balance between putatively beneficial and potentially pathogenic microbial communities. The mechanistic link between root exudates and beneficial microbe recruitment—implied by our Mantel test correlations—has been recently elucidated in cucumber *Fusarium* wilt systems. A study demonstrated that resistant cucumber cultivars exhibit distinct root exudate profiles enriched in defensive compounds, which correlate with the enrichment of beneficial bacteria, including *Streptomyces*, *Cellvibrio*, and *Ensifer* [14]. Moreover, exogenous application of specific metabolites to susceptible cultivars significantly suppressed wilt disease by restructuring rhizosphere bacterial communities. These findings suggest that the metabolite-mediated recruitment of beneficial bacteria—such as the *Sphingomonas* enrichment observed in pepper continuous cropping [15]—represents a conserved mechanism that may be harnessed to mitigate continuous cropping obstacles.

Future studies should investigate whether targeted interventions can reverse these shifts and mitigate continuous cropping obstacles. The co-application of PGPR and organic amendments has been shown to effectively reduce *Fusarium oxysporum* abundance in tomato continuous cropping soil while increasing beneficial *Bacillus* and *Pseudomonas* populations [67]. Similar strategies of constructing composite microbial consortia have been successfully applied in environmental remediation, suggesting the potential of tailored microbial communities for agricultural systems [68]. Previous studies have demonstrated that *Bacillus*-based biofertilizers can effectively restructure rhizosphere microbial communities and mitigate continuous cropping obstacles in other perennial plant systems [69]. Similarly, tomato residue retention under high-temperature treatment promoted colonization of heat-tolerant beneficial biocontrol microbes (*Bacillus*, *Chaetomium*, *Mycothermus*) without *Fusarium* enrichment, as revealed by redundancy analysis and Mantel tests [70].

However, several critical validations are needed for our specific system: (i) confirmation that the enriched *Fusarium* taxa include pathotypes pathogenic to celery requires species-level identification or pathogenicity testing; (ii) the link between *Bacillus* depletion and loss of soil suppressiveness requires bioassays or disease incidence data; (iii) the functional significance of phylum-level shifts (e.g., *Mortierellomycota* decline) requires metagenomic, metatranscriptomic, or enzymatic validation, as phylum-level inference alone is insufficient to establish functional loss; and (iv) the role of unmeasured variables (root exudates, autotoxic compounds, physical properties) should be addressed in future controlled experiments.

Collectively, our findings support an emerging conceptual framework in which continuous cropping obstacles are associated with systemic rhizosphere microecological reorganization rather than simple nutrient depletion. As articulated in recent reviews, long-term monoculture imposes sustained selective pressures on the rhizosphere, resulting in directional shifts in microbial community structure: reduced microbial diversity, depletion of functionally beneficial taxa (e.g., *Bacillus*), and enrichment of host-adapted pathogens (e.g., *Fusarium*) [15,50]. However, we emphasize that this framework remains hypothetical and requires experimental validation. This bacterial suppression-fungal proliferation pattern, coupled with altered nutrient-microbe coupling relationships, may represent a conserved response across diverse agricultural systems—from vegetables and fruits to medicinal plants and edible fungi [23]—but direct confirmation of causality in each system is essential.

## 5. Conclusions

This study compared soil physicochemical properties, microbial diversity, and community composition between continuous cropping (CC, four consecutive celery cycles) and non-continuous cropping (CK, first planting after a fallow period) systems. CC was associated with severe depletion of available nitrogen and potassium (AN −65.9%, AK −69.9%) and a modest but statistically significant increase in total phosphorus (+8.0%) alongside reduced available phosphorus (−14.1%). CC coincided with diametrically opposed diversity responses: bacterial α-diversity and richness decreased significantly, whereas fungal diversity and richness more than doubled. Genera that include many plant growth-promoting rhizobacteria (e.g., *Bacillus*, −89.3%; *Pseudomonas*, −30.8%) were markedly depleted in CC soils, while the genus *Fusarium*, which includes the celery wilt pathogen *F. oxysporum* f. sp. *apii*, showed a 10.9-fold enrichment. Mantel tests revealed statistically significant correlations between specific soil properties (TP, AN, OC for bacteria; TP, TK, AP, AN for fungi) and microbial community structure, pointing to potentially important environmental filters.

Collectively, these findings suggest that continuous celery cropping was associated with a shift from a bacterial-dominated to a fungal-enriched profile, accompanied by depletion of genera that include many PGPR strains and enrichment of the genus *Fusarium*. However, several critical limitations must be emphasized: our study is correlational and does not demonstrate causation; genus-level functional inference is a simplification (PGPR is a strain-level trait, and not all *Fusarium* species are pathogenic); all taxonomic shifts are reported as relative abundances; and the proposed links to soil suppressiveness or disease risk remain hypotheses requiring experimental validation (e.g., inoculation trials, pathogenicity tests, or functional profiling). Despite these limitations, the observed patterns provide a theoretical basis for developing testable hypotheses and targeted interventions, such as *Bacillus*-based biofertilizers, nutrient management to restore AK and AN levels, and crop rotation to mitigate continuous cropping obstacles.

## Figures and Tables

**Table 1:** Soil physicochemical properties of continuous cropping (CC) and control (CK) treatments.

	TN g/kg	TP g/kg	TK g/kg	AP mg/kg	AK mg/kg	AN mg/kg	OC g/kg	pH
CC	1.08 ± 0.017 B	2.19 ± 0.0058 A	5.19 ± 0.044 B	36.47 ± 0.74 B	130.67 ± 4.041 B	49.17 ± 1.67 B	14.57 ± 0.058 B	7.82 ± 0.040 a
CK	1.48 ± 0.032 A	2.027 ± 0.015 B	6.81 ± 0.061 A	42.47 ± 1.93 A	433.67 ± 5.51 A	144.33 ± 5.86 A	22.37 ± 0.32 A	7.89 ± 0.040 a

Data are presented as mean ± SD (*n* = 3 biological replicates, each measured in triplicate). Different uppercase letters indicate significant differences at *p* < 0.01; different lowercase letters indicate *p* < 0.05. CC: continuous cropping (four consecutive cycles). CK: control (first celery planting after one season of fallow).

**Table 2:** The diversity and richness indices of bacterial and fungal communities.

	Sample	ACE	Chao	Sobs	Simpson	Shannon	Coverage
Bacterial	CC	2018.25 ± 23.64 B	1961.079 ± 21.76 B	1809.00 ± 7.21 B	0.003 ± 0.0001 A	5.66 ± 0.078 B	0.9895 ± 0.0004 a
	CK	2622.97 ± 92.35 A	2531.53 ± 78.40 A	2402.33 ± 56.32 A	0.019 ± 0.0030 B	6.67 ± 0.018 A	0.9883 ± 0.0013 a
Fungal	CC	619.22 ± 74.66 A	613.91 ± 70.89 A	591.00 ± 59.10 A	0.042 ± 0.0038 B	4.13 ± 0.077 A	0.9998 ± 0.0001 a
	CK	295.99 ± 25.34 B	295.088 ± 24.63 B	292.00 ± 22.27 B	0.071 ± 0.0052 A	3.34 ± 0.059 B	0.9991 ± 0.0004 a

Data are presented as mean ± SD (*n* = 3). Uppercase letters indicate *p* < 0.01; lowercase letters indicate *p* > 0.05.

## Data Availability

The raw sequencing data generated in this study have been deposited in the NCBI Sequence Read Archive (SRA) and are publicly available under BioProject accession number PRJNA1453622. The dataset is fully accessible without any restrictions.
